# Detecting the Pre-Disease State of Single Sample Through the Change in Local Network Enrichment Level

**DOI:** 10.3390/genes17040425

**Published:** 2026-04-03

**Authors:** Zhenshen Bao, Ying Wang, Zhiyu Liu, Xianbin Li, Yunfei Bai

**Affiliations:** 1 School of Information Engineering, Taizhou University, Taizhou 225300, China; 2 State Key Laboratory of Bioelectronics, School of Biological Science and Medical Engineering, Southeast University, Nanjing 211189, China; 3 College of Rehabilitation and Physical Education, Nanjing Normal University of Special Education, Nanjing 210038, China; 15195986641@163.com; 4 School of Computer and Big Data Science, Jiujiang University, Jiujiang 332000, China; lixb88@gzhu.edu.cn

**Keywords:** pre-disease state, dramatic shift, single sample, enrichment level, local network

## Abstract

**Background**: In complex biological processes, there exists a tipping point (pre-disease state) when the system undergoes a sudden and dramatic shift to a contrasting state. Accurate detection of the pre-disease state is critical for preventive medicine. However, precise detection of the pre-disease state proves challenging due to the clinical single-sample problem. **Methods**: To address this challenge, in this study, we introduce a novel single-sample pre-disease state detection method based on the change in local network enrichment level. **Results**: We validated the proposed method on five independent real datasets, including one influenza virus infection time-course dataset and four tumor datasets. Experimental results confirmed that the proposed method can accurately identify the pre-disease state prior to overt disease onset. Further analysis verified key genes identified by the proposed method in pre-disease state are associated with viral infection and immune dysregulation for the influenza dataset, and tumor metastasis for the tumor datasets. **Conclusions**: These results demonstrate that this method is a robust and biologically interpretable tool for single-sample pre-disease state detection, with great potential for clinical translation in individualized preventive medicine.

## 1. Introduction

Based on the core assertions of bifurcation theory and critical slowing down theory, a tipping point exists in the evolution of a complex system, which is a key juncture marking a sudden transition from the original stable state to a distinctly different new state [1,2]. As a typical complex system, a biological system also follows this law in its dynamic evolutionary processes, and this theoretical framework provides an important perspective for deciphering the intrinsic mechanisms of disease occurrence and development. Thus, along the trajectory of individual disease progression, the corresponding biological system undergoes three states: a normal state, a pre-disease state, and a disease state. The normal state means that an individual is healthy at this state. And the disease state indicates that an individual has got sick at this state. The pre-disease state is a critical transitional phase connecting healthy homeostasis and the disease state. At this stage, the biological system will be with low stability, and its self-repair capacity has weakened. With timely targeted intervention, the system can revert to healthy homeostasis; conversely, in the absence of effective interventions, the system will cross the critical threshold and irreversibly slide into the disease state. It is evident that the accurate identification of the pre-disease state is the core breakthrough point for the prevention and control of malignant diseases.

A biological system can be modeled as a complex regulatory network composed of thousands of interacting genes, whose coordinated expression patterns govern the functional stability and dynamic responses of system. With the rapid advancement of high-throughput technologies, researchers can now simultaneously capture comprehensive gene expression signatures across the entire transcriptome. This technological leap has empowered us to investigate the state transitions of biological systems during disease progression from a molecular perspective. However, as the pre-disease state is defined as the limiting phase of the normal state, its global gene expression profile is often highly similar to that of the normal state. To address this bottleneck, Chen et al. proposed the dynamic network biomarker (DNB) theory [1,2], which selects a small subset of genes called DNBs using the change in correlations between the DNBs and standard deviations of DNB. Despite its theoretical innovation and promising performance in preclinical studies, the DNB method faces a significant practical limitation in clinical settings. The calculation of correlations and standard deviations require multiple measurements collected from the same individual at each time point to capture the accurate and precise variability of the biological system. However, in clinical practice, for an individual, we usually can only acquire one testing sample at a time point. This severely restricts its applicability in real-world clinical scenarios.

This limitation highlights the need for further methodological advancements to adapt DNB theory for single-sample analysis. A diverse array of computational approaches has been proposed in recent years, each leveraging unique statistical, network-based, or distributional properties to capture early warning signals. In the pre-disease state, DNB genes typically show a bimodal distribution, indicating system transitional instability. This shifts to a unimodal distribution in the stable normal state. To quantify this distributional shift, Chen et al. developed a probabilistic approach called DNB-S using the Kullback–Leibler (KL) divergence [3]. Building on similar distributional insights, Zhong et al. and Yan et al. proposed sKLD [4] and sJSD [5] methods, which quantified disturbance caused by a case sample relative to the reference samples by transforming the gene expression data to cumulative Gaussian probability using KL divergence and JS divergence, respectively. Other strategies focus on estimating gene network perturbations or deviations from a reference state to enable single-sample DNB identification. Liu et al. proposed sDNB, a perturbation-based method that assesses gene expression deviations in an individual case sample by comparing it to a set of n matched normal reference samples [6]. Yu et al. proposed iENA to extract high-order statistical and dynamical information from omics data [7]. Liu et al. further advanced this direction with two complementary methods: l-DNB which calculates DNB scores for the first-order local network of each gene to reflect single-sample network dynamics [8], and SLE which quantifies how an individual sample perturbs local network correlations relative to a reference group [9]. Zhong et al. introduced sNMB, which quantifies statistical disturbances in a case sample against relatively healthy samples by evaluating changes in both gene expression variance and correlation [10] while their subsequent SPNE method measures perturbation of directed gene networks using network entropy [11]. The CPMI method incorporates case samples into reference-based calculations of perturbed neighborhood mutual information to track real-time system changes [12]. Peng et al. proposed the DNFE method, that leverages connectivity variation in local time-specific directed networks to detect the system state change [13]. Notably, parallel lines of research have explored alternative strategies that mainly investigate the changes in the features of a gene regulatory network. Huo et al. identified signal perturbation in disease-related pathways as a predictor of pre-disease states [14], while their later work quantified variations in gene–gene interaction strength across the entire regulatory network to detect the pre-disease state [15]. Bao et al. proposed two complementary approaches: one capturing variations in the neighboring expression features of individual genes [16], and another predicting pre-disease states based on coordinated changes in gene expression ranks within a network [17]. Despite significant progress, these methods have notable limitations. Many rely on Pearson’s Correlation Coefficient (PCC) for gene–gene correlation shifts and standard deviation for expression variability, which can be inaccurate with small reference samples common in clinical scenarios. Furthermore, these methods often ignore the higher-level semantics of the DNBs, such as the functional clustering.

The gene set enrichment analysis (GSEA) method can enable robust quantification of the enrichment level of differentially expressed genes (DEGs) within functionally coherent gene sets, which composed of highly correlated genes that collectively drive biological processes [18]. Inspired by this and the DNB theory, in this study, we developed a novel single-sample pre-disease state detected algorithm based on the shifts in **L**ocal **N**etwork **E**nrichment level, called **LNE**. Specifically, a local network for a gene is constructed by itself and its first-order neighboring genes, and is seen as a gene set used in GSEA. Subsequently, the enrichment level of this local network is computed following the calculation process of GSEA. LNE methods are applied on five real datasets to demonstrate their abilities in the acute detection of the pre-disease state. The results consistently demonstrated that LNE can reliably detect the pre-disease state in individual samples prior to the onset of overt disease. The results of GO BP terms/KEGG pathway enrichment analysis for the influenza virus infection dataset further confirmed the biological relevance of LNE’s outputs. For the four tumor datasets, the LNE-identified DNBs are strongly linked to tumor metastasis. Collectively, these findings underscore the superior performance of LNE in sensitivity and specificity. Hence, the proposed approach holds immense potential for clinical applications as it enables the accurate identification of pre-disease states from a single sample.

## 2. Materials and Methods

### 2.1. Data and Data Processing

To demonstrate the practical utility of the proposed LNE method, we first acquired a real time-course gene expression dataset of influenza A virus H3N2 infection (GSE30550) from the Gene Expression Omnibus (GEO) database (https://www.ncbi.nlm.nih.gov/geo/, accessed on 12 September 2025). This dataset was profiled on the Affymetrix GeneChip Human Genome U133A 2.0 Array, a microarray platform covering most human protein-coding genes via specific probe sets. For the validation of the H3N2 dataset results, an influenza A virus H1N1 infection dataset (GSE36553) was additionally employed, which comprises 5 control (baseline) samples and 6 infected samples at each subsequent time point. This dataset was profiled on the Illumina HumanHT-12 V3.0 expression beadchip platform, which provides high-throughput transcriptome profiling with broad coverage of human protein-coding genes. For preprocessing of the two datasets, multiple probes mapping to the same gene were consolidated by taking the mean of their expression values as the final expression level for the corresponding gene; probes without a matched NCBI Entrez gene symbol were excluded from further analysis.

Four tumor datasets were retrieved from The Cancer Genome Atlas (TCGA) database (http://cancergenome.nih.gov, accessed on 9 October 2025), including breast cancer (BRCA), esophageal carcinoma (ESCA), rectum adenocarcinoma (READ), and stomach adenocarcinoma (STAD). These TCGA datasets were generated using high-throughput RNA-seq technology with genome-wide transcriptomic coverage of coding and non-coding RNA profiles. Each dataset contained both tumor tissue samples and paired tumor-adjacent normal tissue samples. Tumor samples were stratified into distinct clinical stages based on the stage annotation information obtained from the TCGA database, and samples with missing clinical stage information were removed from the dataset to ensure data integrity. Detailed characteristics of all the aforementioned datasets are summarized in Table 1.

In this study, different from the single-sample datasets, the four tumor datasets are with no individual-based samples across all time points. And the tumor datasets are used to demonstrate that our method can capture state-associated expression trends across tumor progression. Therefore, for the four datasets, the expressions of genes are obtained by their average value in each stage. Then, for each dataset, such data with mean gene expression at each stage can be seen as a single-sample data.

### 2.2. Algorithm to Reveal the Critical State Based on LNE

According to the DNB theory [1,2], when the system is in the pre-disease state, there exists a group of genes with huge expression changes and highly associated with each other. In a protein and protein interaction (PPI) network, a gene and its first-directed neighbor genes are always highly associated with each other. Thus, to determine whether a gene is a member of DNBs, it is sufficient to examine its differential expression level and the enrichment level of a gene set composed of it and its first-directed neighbor genes in a PPI network. In this study, the GSEA method is employed to quantify the enrichment level of differentially expressed genes for such gene sets [18], and the temporal difference value in gene expression is employed to measure the expression variations of these genes.

**Figure 1:**
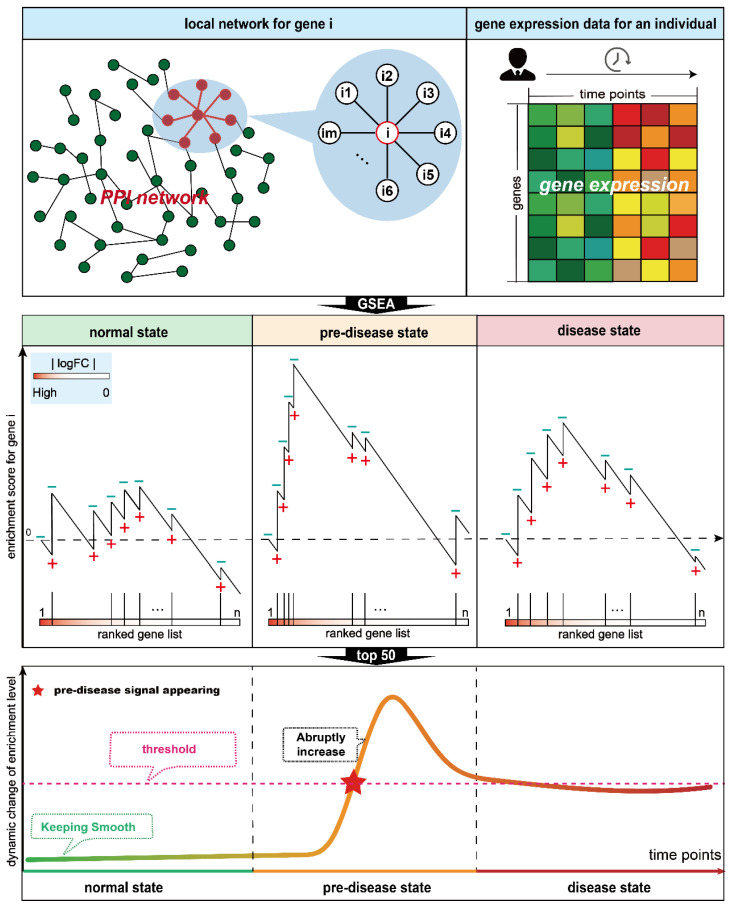
The schematic illustration of LNE.

For a gene i in the PPI network, N is downloaded from the STRING database, and a local gene set ${\mathrm{GS}}_{i}$ can be extracted, which consists of the gene and its 1st-order neighbor genes. Then, we carry out the following algorithm to identify the pre-disease state by using gene expression data from only one individual (See Figure 1).

[Step 1] Given *n* samples from the healthy or relatively healthy time points, the baseline expression value $E_{i}^{b}$ of gene i can be calculated as follows:

(1) $$E_{i}^{b}=mean\left(E_{i}^{1},E_{i}^{2},\ldots ,E_{i}^{n}\right)$$

where $E_{i}^{1},E_{i}^{2},\ldots ,E_{i}^{n}$ are the expression values of gene i in the *n* normal samples, respectively. For the time-course gene expression dataset which has included the relative normal data from *n* initial time points, the expression baseline will be calculated based on these data. If there are no such data, the expression baseline derived from healthy samples will be built using publicly available normal datasets. For a time point *t*, the expression variation $\Delta E_{i}^{t}$ of gene i is calculated as follows:

(2) $$\Delta E_{i}^{t}=\left|E_{i}^{t}-E_{i}^{b}\right|$$

where $E_{i}^{t}$ is the expression values of gene i at the time point *t*.

[Step 2] For a time point *t*, rank order all genes in the network *N* to form $L_{t}$ according to their expression variation at that time point. An enrichment score ${\mathrm{ES}}_{i}^{t}$ is calculated to evaluate the fraction of genes in a local gene set ${\mathrm{GS}}_{i}$ (“hits”) weighted by their correlation and the fraction of genes not in this set (“misses”) present up to a given position in $L_{t}$.

(3) $$P_{\mathrm{hit}}^{t}\left({\mathrm{GS}}_{i}\right)=\sum_{j\in {\mathrm{GS}}_{i}}\frac{\Delta E_{j}^{t}}{N_{R}^{t}}$$

(4) $$N_{R}^{t}=\sum_{j\in {\mathrm{GS}}_{i}}\Delta E_{j}^{t}$$

(5) $$P_{\mathrm{miss}}^{t}\left({\mathrm{GS}}_{i}\right)=\sum_{j\notin {\mathrm{GS}}_{i}}\frac{1}{{N-N}_{H}^{i}}$$

where $\Delta E_{j}^{t}$ is the expression variation of gene j at the time point *t*. $N_{H}^{i}$ is the number of genes which are in the gene set ${\mathrm{GS}}_{i}$ and $L_{t}$ simultaneously. The enrichment score ${\mathrm{ES}}_{i}^{t}$ is the maximum deviation from zero of $P_{\mathrm{hit}}^{t}-P_{\mathrm{miss}}^{t}$. For a randomly distributed gene set, such enrichment score will be relatively small, but if it is concentrated at the top or bottom of the list, or otherwise non-randomly distributed, the score will be correspondingly high.

[Step 3] The enrichment level of a gene set ${\mathrm{GS}}_{i}$ at the time point *t* is represented by the significance of the observed ${\mathrm{ES}}_{i}^{t}$ by comparing it with the set of scores computed with randomly assigned phenotypes.

1. Randomly assign the original phenotype labels to samples, reorder genes, and re-compute a random enrichment score.
2. Repeat step 1 for 1000 permutations, and create a histogram of the corresponding enrichment score.
3. Estimate nominal *p* value $P_{i}^{t}$ by using the positive or negative portion of the distribution corresponding to the sign of the observed ${\mathrm{ES}}_{i}^{t}$.
4. Calculate the enrichment level ${\mathrm{EL}}_{i}^{t}$ based on the *p* value $P_{i}^{t}$.

(6) $${\mathrm{EL}}_{i}^{t}=-\log_{10}\left(P_{i}^{t}\right)$$

[Step 4] the enrichment level change $\Delta {\mathrm{EL}}_{i}^{t}$ of the gene i at the time point *t* is calculated, i.e.,

(7) $$\Delta {\mathrm{EL}}_{i}^{t}=\left|{\mathrm{EL}}_{i}^{t}-{\mathrm{EL}}_{i}^{1}\right|$$

where ${\mathrm{EL}}_{i}^{t}$ and ${\mathrm{EL}}_{i}^{1}$ are the expression ranking of gene $g_{i}$ at the time point *t* and the first time point, respectively. The local LNE score $S_{i}^{t}$ of the gene i at the time point *t* can be gained by combining the expression change $\Delta E_{i}^{t}$ and enrichment level change $\Delta {\mathrm{EL}}_{i}^{t}$, i.e.,

(8) $$S_{i}^{t}=\Delta E_{i}^{t}\cdot \Delta {\mathrm{EL}}_{i}^{t}$$

According to the DNB theory, DNBs typically consist of a small set of genes that exhibit dramatic expression fluctuations and strongly functionally interact with each other [2]. Thus, for a time point, the top *m* (*m* = 50) genes with the largest score $S_{i}^{t}$ can be seen as the candidate DNBs. Then, the system state at the time point *t* can be represented as the LNE score $S^{t}$ which is calculated based on these 50 genes, i.e.,

(9) $$S^{t}=\frac{\sum_{i=1}^{m}S_{i}^{t}}{m}$$

When the system is near the pre-disease state, the expression change $\Delta E_{i}^{t}$ and enrichment level change $\Delta {\mathrm{EL}}_{i}^{t}$ of these DNB biomolecules will suddenly increase. Accordingly, the system variation score $S^{t}$ will sharply increase. If there is a sufficient number of normal samples, to identify such a sharp increase in variation score, a threshold θ is determined from the system variation score and $S^{t}$ of the normal samples using Z-test following a standard normal distribution. The score corresponding to the upper 0.05 is set as the threshold. For the dataset with only one normal sample, the abrupt increase in LNE scores means the pre-disease state appears. For the dataset without a normal sample, the threshold can be derived from a general tissue-matched healthy reference or a consolidated normal expression profile.

### 2.3. Functional Analysis

In this study, we designate the top 50 genes with the highest system variation scores at the pre-disease state identified via the LNE algorithm as individual-specific key genes, hereafter referred to as *LNE* genes. These *LNE* genes, which are prioritized for their robust perturbation signals during the critical pre-disease transition phase, are subsequently subjected to functional enrichment analyses of Gene Ontology Biological Process (GO BP) terms and Kyoto Encyclopedia of Genes and Genomes (KEGG) pathways using DAVID (Database for Annotation, Visualization, and Integrated Discovery), a widely used web-based bioinformatics analysis tool accessible at https://davidbioinformatics.nih.gov/ [19,20].

## 3. Results

### 3.1. Identifying the Pre-Disease State During Influenza Virus Infection

To illustrate the computational effectiveness of the proposed LNE method, a real single-sample dataset, influenza virus H3N2 infection data, which contains 17 subjects and 16 time points, is employed. For such a dataset, the successful identification of influenza virus H3N2 infection before symptoms appear demonstrates the effectiveness and accuracy of the proposed LNE method.

**Figure 2:**
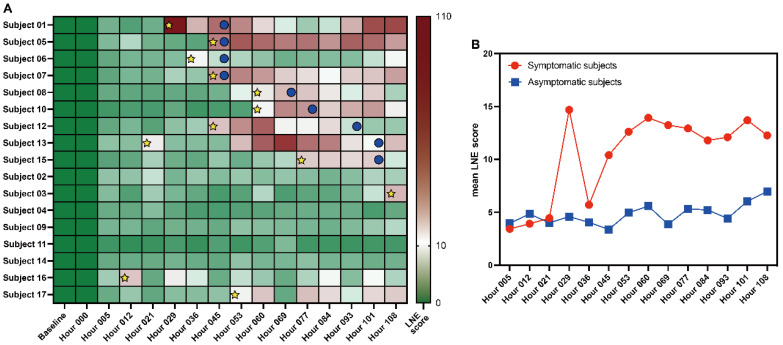
The summarized prediction results of the influenza virus infection dataset. (**A**): The heatmap for datasets GSE30550, the time points with yellow star marks are the pre-disease state appearing time point, and the time points with blue circle marks are the symptom appearing time points; (**B**): the average LNE scores across all symptomatic subjects for the dataset GSE30550.

As shown in Figure 2A, for each subject, the time points with yellow star marks are the pre-disease state appearing time points predicted by LNE method, and the time points with blue circle marks are the symptom appearing time points. For each symptomatic subject, the LNE score increased significantly (exceeding the threshold θ=12) at a time point no later than symptom onset. Such results mean that the proposed LNE method can identify the pre-disease state for a single sample. For most asymptomatic subjects, the LNE scores are lower than the threshold at all time points. In addition, for asymptomatic subjects 3, 16, and 17, early warning signals can be detected, which may indicate that the three subjects had symptoms after the experiment ended. Figure 2B shows the mean LNE scores for symptomatic subjects and asymptomatic subjects at each time point. The mean LNE scores of the symptomatic subjects are relatively higher and more unstable than that of the asymptomatic subjects. The above results demonstrate that the LNE score can predict the pre-disease state just before the onset of influenza symptoms appearing.

### 3.2. Identifying the Pre-Disease State During Tumor Progression

For the tumor datasets, each tumor adjacent (TA) sample is used to calculate baseline change in enrichment score. And because there are no individual-based samples across whole stages, the expressions of genes are obtained by their average value in each stage. Then, for each tumor dataset, such data with mean gene expression at each stage can be seen as single-sample data. Metastasis is the primary cause of most cancer-related fatalities and represents the ultimate challenge in our battle against cancer as a life-threatening disease [21]. The cancer may spread from primary site to adjacent lymph nodes at Stage II [22]. Thus, for these tumor datasets, the pre-disease state identified by the proposed LNE method should be earlier than Stage III.

**Figure 3:**
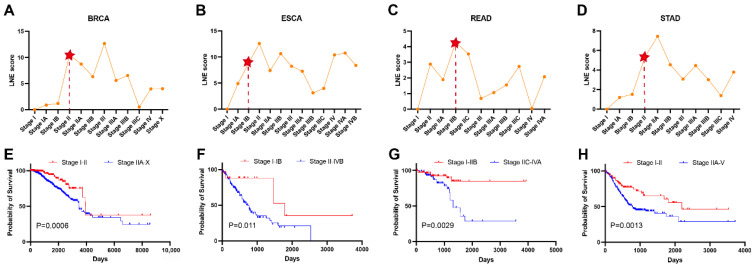
Identification of the pre-disease state of tumor near metastasis in three cancers. (**A**–**D**): Identifying the pre-disease state for (**A**): BRCA, (**B**): ESCA, (**C**): READ, and (**D**): STAD, and the stages with red star marks are the pre-disease state appearing time points. (**E**,**F**): Comparing survival curves between the before and after pre-disease state in (**E**): BRCA, (**F**): ESCA, (**G**): READ, and (**H**): STAD.

As shown in Figure 3A–D, the stages with red star marks are the pre-disease state appearing time points predicted by the LNE method. According to the calculation of threshold, multiple LNE scores from normal samples are needed. Thus, for these tumor datasets, the threshold cannot be acquired. The abrupt increase in LNE scores for each tumor dataset means the tumor metastasis (pre-disease state) appears. For the BRCA dataset, as shown in Figure 3A, the LNE score increases significantly from Stage IB to II, which indicates the extension of tumors into adjacent lymph nodes at Stage II. Figure 3E shows that the survival time of samples from the normal state (Stages I to II) is significantly longer (*p* = 0.0006) than that of samples from the disease state (Stages IIA to X). When applied to the ESCA dataset, as seen in Figure 3B, the drastic increase in LNE score appearing at Stage IB also suggests that nearby metastasis occurs after Stage II. As shown in Figure 3F, there is a significant difference (*p* = 0.011) between the survival time of samples taken before and after the identified pre-disease state (Stage IB). As shown in Figure 3C, the LNE scores rose significantly from Stage IIA to IIB, representing that the tumor cells metastasize to nearby tissues/organs at Stage II and distant tissues/organs at stage IV. It is also seen from Figure 3G that the survival time of samples from Stages I-IIB is significantly longer (*p* = 0.0029) than that of samples from Stages IIC–IVA. For the STAD dataset, as seen in Figure 3D, there is a significant increase from Stages IB to II in the LNE score. This indicates that after Stage IIB, the tumor will invade the adjacent serosal layer of the gastric wall and eventually metastasize to distant sites. Furthermore, the survival time of the samples in Stage I–II is significantly longer (*p* = 0.0013) than that of samples in Stage IIA–V. The above results demonstrate that the LNE method can identify the pre-disease state just before the onset of tumor metastasis.

### 3.3. Revealing Potential Biological Functions of Common LNE Genes for Influenza Dataset

There are 29 common *LNE* genes shared among more than four symptomatic subjects. The detailed *LNE* genes for each symptomatic subject are listed in Supplementary Table S1. To investigate the molecular regulation mechanism, a PPI network and KEGG signaling pathway enrichment analysis are performed based on these genes.

**Figure 4:**
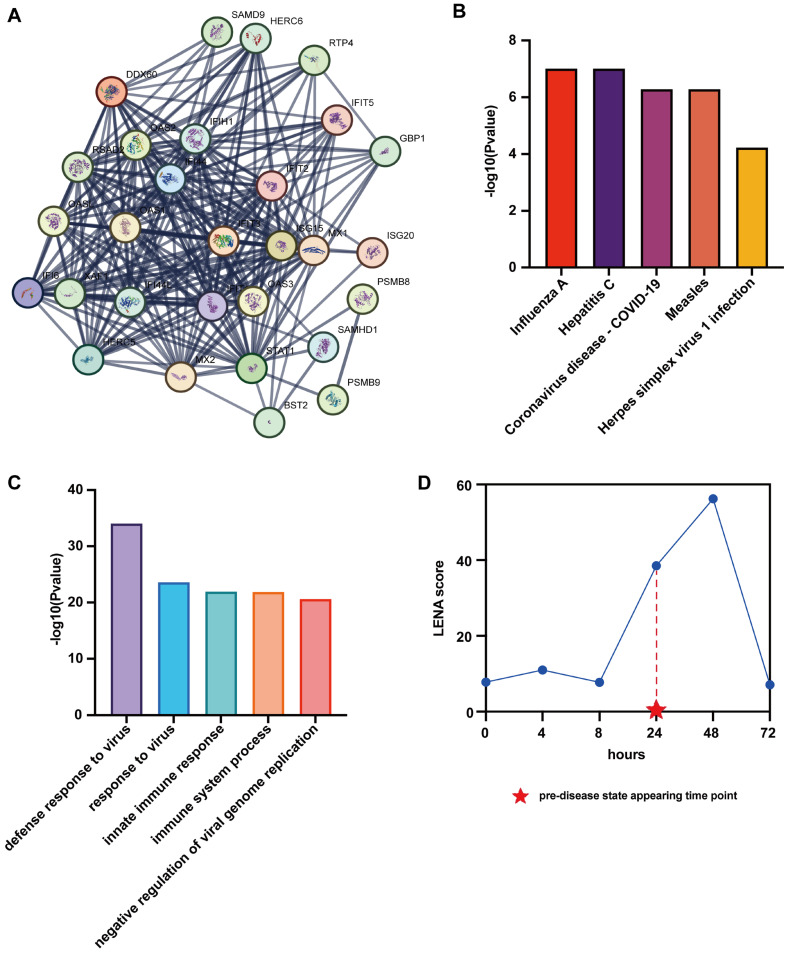
The results of functional analysis for the 29 common *LNE* genes identified using influenza infection dataset. (**A**): the local PPI network construct by the 29 common *LNE* genes; (**B**): the top 5 enriched KEGG pathways of these genes with the smallest FDR adjusted *p*-values; (**C**): the top 5 enriched GO BP terms of these genes with the smallest FDR adjusted *p*-values; (**D**): performance of the LNE method in GSE36553 using the identified *LNE* genes.

For the PPI network analysis, these genes are uploaded to the STRING database to construct a local PPI network with a confidence level of 0.80. As shown in Figure 4A, the constructed local PPI network consists of 29 nodes and 237 edges. The average node degree of the local PPI network is 16.3, while the average local clustering coefficient is found to be 0.87. These results indicate that these *LNE* genes are highly correlated to each other.

For the KEGG signaling pathway enrichment analysis, these genes are uploaded to DAVID dataset. The top five pathways with the smallest *p*-values (FDR) are shown in Figure 4B, including “Influenza A”, “Hepatitis C”, “Coronavirus disease—COVID-19”, “Measles”, and “Herpes simplex virus 1 infection”. Pathway “Influenza A” is the pathway which is mainly associated with influenza virus infection. The remaining pathways are related to different virus infections. This means that these *LNE* genes are associated with the virus infection. To validate such results, GO BP enrichment analysis is employed based on them. As shown in Figure 4C, the top five BP GO terms with the smallest *p*-values (FDR) are all related to cellular immune response for virus infection. Such results indicate that these *LNE* genes can be seen as the DNBs of influenza virus infection.

To further investigate the molecular regulation mechanism of these *LNE* genes, the dataset GSE36553 which mainly studies the temporal influenza virus infection is used to evaluate the LNE method based on these 29 genes. Different from the single-sample datasets, there are no individual-based samples across all time points in this dataset. Thus, the LNE score based on 29 *LNE* genes is calculated for each sample according to the procedure of the proposed method. Then, for each time point, the mean LNE score is used to identify the pre-disease state for the influenza virus infection. The abrupt increase in the mean LNE score at a time point means that most samples at this time point are in the pre-disease state. This indicates that these 29 *LNE* genes can be used as markers of the pre-disease state for influenza virus infection, and also demonstrates the effectiveness of the proposed LNE method. As shown in Figure 4D, the LNE score drastically increases from the 8- to 24-h time point. This indicates that the early warning signal appears at the 24-h time point, while the released infectious virus load peaked at the 48-h time point in clinical experiment [23]. Therefore, for such a dataset, our method can successfully detect the early warning signal of the pre-disease state for influenza virus H1N1 infection based on the 29 *LNE* genes detected by the dataset GSE30550.

### 3.4. Revealing Potential Biological Functions of Common LNE Genes for Tumor Datasets

For the four tumor datasets, there are 11 common *LNE* genes shared among more than three tumor datasets (shown in Table 2). The detailed *LNE* genes for each tumor dataset are listed in Supplementary Table S2. These genes are associated with tumor metastasis. For instance, *ADH1B* is a potential therapeutic target, offering novel strategies to address the challenges of metastasis in LUAD [24]. *ASPM* serves as a crucial oncogenic part in regulating LUAD cell migration [25]. And METTL3—mediated m6A methylation of *ASPM* can drive hepatocellular carcinoma cells metastasis [26]. *BUB1* acts as a positive regulator of gastric cancer cell metastasis by activating the TRAF6/NF-κB/FGF18 pathway through METTL3-mediated m6A methylation [27]. *BUB1B* promotes ovarian cancer cell metastasis by activating the wnt/β-catenin pathway [28]. USP4-mediated *CENPF* upregulation was a critical regulator of metastasis of colorectal cancer [29]. Overexpression of *CENPF* correlates with poor prognosis and tumor bone metastasis in breast cancer [30]. High *COL10A1* mRNA expression is associated with gastric cancer metastasis [31]. *KIF14* is a potential oncogene and is involved in the metastasis of various cancers [32]. *MAGEA3* expression facilitates cervical cancer cell metastasis via actuating wnt signaling pathway [33]. *MAGEA6* can positively regulate *MSMO1* and promote migration and invasion of esophageal cancer cells [34]. *NUF2* can drive cholangiocarcinoma migration via inhibiting autophagic degradation of *TFR1* [35]. Upregulated *TPX2* expression contributes to promote migration of breast cancer cells [36].

## 4. Discussion

The accurate identification of the pre-disease state is a pivotal frontier in preventive medicine and precision healthcare. It enables the capture of early, reversible biological perturbations preceding overt disease onset. Thus, it lays the foundation for the development of targeted preventive strategies and ultra-early therapeutic interventions that can halt or reverse disease progression before the emergence of irreversible pathological damage. A major breakthrough in this field came with the proposal of dynamic network biomarker (DNB) theory by Chen et al. which established a framework for identifying pre-disease states by characterizing the dynamic instability of biological regulatory networks [1,2]. Despite its theoretical innovation and proven efficacy in preclinical research, the traditional DNB method is inherently limited by its reliance on multi-sample datasets, a constraint that directly conflicts with the realities of clinical practice. To address this critical translational gap and advance the clinical applicability of DNB theory, the present study developed a novel single-sample pre-disease state detection algorithm, LNE, which leverages variations in local gene network enrichment levels to capture the early warning signals of biological system state transitions from individual gene expression datasets. Although we have shared a similar method for disease early warning detection [17], the proposed LNE method makes a key improvement: different from the previous analytical pipeline that relied on gene-level statistics, the newly developed LNE indicator is optimized specifically for both gene- and network-level statistics. The introduction of network-level statistics can effectively reduce the noise sensitivity of the method and make the results more robust.

To evaluate the performance, robustness, and biological relevance of the LNE method, we conducted comprehensive validation on five independent real-world datasets, including a time-course influenza virus infection dataset (GSE30550) and four tumor datasets from The Cancer Genome Atlas (TCGA) covering breast cancer (BRCA), esophageal carcinoma (ESCA), rectum adenocarcinoma (READ), and stomach adenocarcinoma (STAD). Across all datasets, the LNE-derived pre-disease state detection results exhibited strong consistency with established clinical and experimental observations of disease progression. Such results confirmed the ability of the LNE algorithm to reliably identify the critical transitional phase before the onset of overt pathological phenotypes. Functional annotation of the *LNE* genes further validated the biological interpretability of the method. For the influenza virus infection dataset, these genes were significantly enriched in biological processes and signaling pathways directly associated with viral entry, replication, and host immune response dysregulation that are well-characterized as early drivers of influenza pathogenesis. For the four TCGA tumor datasets, the LNE-prioritized key genes were uniformly linked to tumor metastasis, a hallmark of malignant progression and a critical early event in cancer development that represents a major target for ultra-early cancer intervention. Collectively, these findings demonstrate that the LNE method achieves high accuracy in single-sample pre-disease state detection while maintaining strong biological relevance.

A core conceptual innovation of the LNE method is its unique integration of DNB theory with gene set enrichment analysis (GSEA), which transforms the problem of pre-disease state identification from a purely network dynamics analysis into a gene set enrichment variation problem. By definition, the pre-disease state represents the terminal, unstable limit of the normal state, meaning individual gene expression values often exhibit only subtle or no detectable differences between these two states when assessed in isolation. This renders single-gene expression analysis ineffective for pre-disease detection, as it fails to capture the systemic network-level perturbations that characterize the pre-disease state. LNE addresses this limitation by harnessing GSEA, a well-validated approach that is inherently sensitive to subtle, coordinated fluctuations in the expression of gene sets rather than isolated gene expression changes. LNE further amplifies these signals by quantifying differential expression values across local gene networks. This dual integration of network dynamics and gene set enrichment variation thus allows LNE to detect the subtle, systemic biological changes.

Despite its promising performance, the LNE method is not without limitations. A primary limitation is the inherent sensitivity of the algorithm to noise in gene expression datasets. The LNE method amplifies subtle pre-disease associated signals and may concurrently amplify random technical or biological noise present in the profiling data. This phenomenon was observed in the influenza virus infection dataset (GSE30550), where the LNE detected an apparent pre-disease state in asymptomatic subjects 3, 16, and 17. While these signals may represent the appearance of pre-disease states they may also indicate that the three subjects had symptoms after the experiment ended. They may also be attributable to LNE-mediated amplification of technical noise. This limitation underscores the need for strategies to enhance the noise robustness of the LNE method in the future. A key solution to this challenge will likely come from advances in high-precision gene sequencing and omics profiling technologies. Methodological refinements, such as integrating noise reduction steps into the LNE workflow could also further mitigate noise amplification. Another limitation is that several clinical factors may affect individual disease trajectories and gene expression profiles. Due to the incomplete metadata of the public datasets adopted in this study, these confounding variables are not included for adjustment in the current analysis. Further studies based on prospective clinical cohorts with detailed clinical information are needed to validate and optimize the LNE approach by integrating multiple clinical covariates. And notably, although the LNE method is framed as a single-sample pre-disease state detection strategy, it does not operate entirely independently of reference cohort information. The current framework still relies on relative normal data from initial time points or healthy historical public datasets to establish baseline backgrounds for network estimation and parameter calibration. Therefore, its single-sample capability should not be interpreted as a fully reference-free design. Further improvements will be required to reduce dependence on external reference data and enhance generalizability across heterogeneous clinical populations.

By enabling accurate, biologically interpretable pre-disease state detection from individual gene expression datasets, the LNE provides a scalable and practical tool for integrating pre-disease screening into routine clinical care. Future research will focus on two key directions: first, optimizing noise robustness of the algorithm through methodological and technological refinements as noted above; secondly, expanding the method to integrate multi-omics data to capture a more comprehensive view of biological system perturbations at the pre-disease state.

## 5. Conclusions

In this study, a novel and robust computational algorithm LNE is designed for the accurate identification of the pre-disease state in complex diseases using single-sample gene expression data. A core conceptual and methodological innovation of the LNE method is that it converts the pre-disease state detection problem into a gene set enrichment analysis task, integrating the statistical rigor of gene set enrichment analysis (GSEA) with the network dynamics-centric core of DNB theory to capture subtle biological perturbations that characterize the pre-disease tipping point. As validated across five independent real datasets, an LNE can accurately detect the pre-disease state with a single-sample dataset, and the LNE identified key genes that are strongly associated with viral infection in influenza infection datasets and with tumor metastasis in cancer datasets. This also means that an LNE can link computational pre-disease signals to well-characterized molecular and cellular disease mechanisms. Hence, the proposed LNE method has great potential in clinical early prevention medicine.

## Figures and Tables

**Table 1:** The details of the real datasets.

Datasets	Hours/Stages	Subjects
GSE30550	Baseline, 0, 5, 12, 21, 29, 36, 45, 53, 60, 69, 77, 84, 93, 101, 108	Sx:9/Asx:8
GSE36553	0 h, 4 h, 8 h, 24 h, 48 h, 72 h	-
BRCA	I, IA, IB, II, IIA, IIB, III, IIIA, IIIB, IIIC, IV, X	-
ESCA	I, IA, IB, II, IIA, IIB, III, IIIA, IIIB, IIIC, IV, IVA, IVB	-
READ	I, II, IIA, IIB, IIC, III, IIIA, IIIB, IIIC, IV, IVA	-
STAD	I, IA, IB, II, IIA, IIB, III, IIIA, IIIB, IIIC, IV	-

Sx: symptomatic subjects; Asx: asymptomatic subjects.

**Table 2:** The 11 common *LNE* genes shared among more than 3 datasets for the 4 tumor datasets.

No.	Gene	No.	Gene
1	*ADH1B*	7	*KIF14*
2	*ASPM*	8	*MAGEA3*
3	*BUB1*	9	*MAGEA6*
4	*BUB1B*	10	*NUF2*
5	*CENPF*	11	*TPX2*
6	*COL10A1*	/	/

## Data Availability

The original contributions presented in this study are included in the article. Further inquiries can be directed to the corresponding authors.

## Supplementary Materials

The following supporting information can be downloaded at: https://www.mdpi.com/article/10.3390/genes17040425/s1, Table S1: *LNE* genes identified using influenza dataset, Table S2: *LNE* genes identified using the four tumor datasets.

## Abbreviations

The following abbreviations are used in this manuscript:

DNB	Dynamic network biomarker
KL	Kullback–Leibler
GSEA	Gene set enrichment analysis
DEG	Differentially expressed gene
PCC	Pearson’s Correlation Coefficient
SD	Standard deviations
PPI	Protein and protein interaction
GEO	Gene Expression Omnibus
TCGA	The Cancer Genome Atlas
BRCA	Breast cancer
ESCA	Esophageal carcinoma
READ	Rectum adenocarcinoma
STAD	Stomach adenocarcinoma
GO BP	Gene Ontology Biological Process
KEGG	Kyoto Encyclopedia of Genes and Genomes
